# Experimental and Numerical Study of Computer Vision-Based Real-Time Monitoring of Polymeric Particle Mixing Process in Rotary Drum

**DOI:** 10.3390/polym16111524

**Published:** 2024-05-29

**Authors:** Junghyun Byun, Kwon Joong Son

**Affiliations:** Department of Mechanical and Design Engineering, Hongik University, Sejong 30016, Republic of Korea; c3314401@mail.hongik.ac.kr

**Keywords:** polymer particle mixing, mixing process monitoring, computer vision, discrete element method, mixing index, drum mixer

## Abstract

In the drum mixing of particulate polymers, segregation may occur. By measuring the mixing status in real time, it is possible to implement corrective measures to prevent separation and improve the efficiency of the process. This study aims to develop and validate a real-time vision system designed to monitor the mixing process of polymeric particles in a rotary drum mixer, employing a novel centroid-based model for determining the mixing index. The proposed centroid-based model is capable of addressing the radial particle segregation issue without the need for extra image-processing procedures like image subdivision or pixel randomization. This innovative approach greatly improves computational efficiency by processing over 68 image frames per second. The new processing method is 2.8 times faster than the gray-level co-occurrence matrix method and 21.6 times faster than the Lacey index approach. This significantly improves real-time monitoring capabilities and enables real-time image processing using only affordable single-board computers and webcams. The proposed vision-based system for monitoring rotary drum mixing has undergone validation via cross-validation using discrete element method simulations, ensuring its accuracy and reliability.

## 1. Introduction

Polymers in particle form find widespread use in various industrial applications such as plastic injection molding, synthetic fiber production, blow molding, and composite material manufacturing. Although most manufacturing processes are based on uniform plastic particles, there are instances where a homogeneous mixture of different plastic particles is required. For example, the mixing quality of particulate materials plays a critical role in plastic injection molding [1], 3D printing based on sintering [2], and 3D printing of fused deposition manufacturing (FDM) using recycled plastics [3]. This underlines the importance of efficient material blending and preparation to ensure the consistency and quality of the polymer product.

Efforts to enhance the mixing processes of polymer particles have followed two primary strategies: the design of the device and its operational features. Modifications to the structure, such as the incorporation of baffles in mixing drums [4], have been examined as a way to inhibit segregation throughout the mixing operation. This study explored the efficacy of single-arm, four-arm, and six-arm baffles in drum mixing and their shape optimization. Optimized four- and six-arm designs have been found to show enhanced mixing capabilities when compared with the single-arm design. From an operational point of view, studies have investigated the effects of the initial particle distribution on mixing performance [5] and the impact of drum tilt and rotational speed on mixing effectiveness [6]. The optimization of particle-mixing processes is a crucial aspect of industrial production. To improve the efficiency of these processes, most scholarly efforts primarily utilize a numerical approach marked by detailed analysis of sequential data related to the mixing index (MI). MI is a measurable indicator of the uniformity of the particle mixture, and its analysis is crucial to understanding the underlying mechanisms responsible for the blending process. However, because of the inherent complexity of tracking individual particle trajectories, the experimental determination of MI is a challenging task. Researchers often employ a numerical approach using the discrete element method (DEM) to mitigate this problem [7,8,9,10,11]. This computational technique offers a precise and manageable means of evaluating the dynamics of particulate matter and the effectiveness of amalgamation in a wide range of industrial contexts. Therefore, DEM simulation is most widely used in analogous investigations, allowing the acquisition of kinematic data and aiding in understanding the underlying mechanisms that govern particulate amalgamation processes.

Research into experimental techniques for MI measurement continues to be pursued, in addition to the use of DEM simulation-based methods for MI computation and analysis. Advancements in a variety of measurement technologies for industrial mixing processes highlight their potential to improve process efficiency and product quality, while also removing the need for manual sampling. These advances pertain to the various methods used to measure the properties of materials in industries and pharmaceuticals. Such methods encompass traditional ones such as power, torque and point property measurements, as well as cutting-edge techniques such as tomographic techniques (for example, electrical tomography, magnetic resonance imaging, X-ray, and gamma ray tomography) and spectroscopic measurement technologies (for instance, near-infrared spectroscopy, fluorescence spectroscopy, and Raman spectroscopy) [12]. Furthermore, there is growing interest in research on image processing-based vision technology with the aim of integrating industrial and pharmaceutical materials [12,13].

This study focuses on the application of vision-based techniques for real-time monitoring of commercial mixers due to their straightforward setup and comparatively low setup costs. An essential prerequisite for MI computation in image processing is the capability to distinguish between particle types based on their color or saturation within the color space. Various computational methods, including the Lacey index, mixing entropy, and Gini coefficient, have been established [14]. These methods employ images that have been binarized to determine the uniformity of the mixture. The gray-level co-occurrence matrix (GLCM) approach has been implemented to compute MI algorithms for processed drum mixing videos by extracting information on texture heterogeneity from changes in the gray levels of adjacent pixels [15,16,17]. Similarly, a vision system that examines segregation phenomena in polymer particle mixing has been developed using a segregation index (SI) similar to Lacey index-based MI [18]. A pixel randomization technique has been suggested to acquire a reference for the ideal mixed state necessary for MI calculation [19]. Most studies on calculating MI based on image-processing algorithms have been carried out with stored experimental or simulated images for subsequent processing. This is because processing images in real time, at a standard video frame rate of 30 frames per second (fps), is quite challenging due to the significant computational load involved in tasks such as image randomization, image subdomain segmentation, and the aggregation of neighboring pixel data. One of the more efficient algorithms for computing mutual information involves a technique based on the relative distance between the centroids of the particles [20,21,22]. However, this technique is hindered by the issue of severe fluctuation in centroid distances along the rotational cycle of rotary mixers.

This paper aims to develop a monitoring system capable of performing real-time MI calculations at image-processing speeds exceeding 30 fps. This goal should be achieved by using an affordable single-board computer (SBC) such as a Raspberry Pi with a compact red-green-blue (RGB) camera module, which will serve as an alternative to expensive vision processing hardware. Therefore, this study avoids computationally demanding MI calculation algorithms that require subimage segmentation or randomization processes. Instead, this research introduces a new centroids-based model for MI calculation that incorporates a sigmoid function and a standard deviation weighting factor. This model is designed to tackle several known problems associated with the centroid-based MI method, such as negative MI values, issues with sensitivity, and erroneous analysis of radial particle segregation. In addition, the monitoring system employs a fast data filter to minimize data oscillations. This methodology ensures system efficiency while significantly improving the accuracy of monitoring the mixing process, even with the constraints of limited computational resources.

To provide a systematic presentation of a highly efficient algorithm for monitoring particle mixing in real time using computer vision processing, this paper is organized as follows. The next section elaborates on the performance of the simulations of the discrete element method to acquire test images, the formulation of a novel centroid-based MI model, and the development of vision-processing algorithms. The following section presents the construction of a drum mixer apparatus equipped with a vision-processing unit to monitor particle mixing and analyze the vision-processing results for experimental data. The final section presents conclusions based on research achievements and discusses the importance of this study, as well as future research directions.

## 2. Computer Vision Process

This section outlines a real-time vision processing algorithm for calculating particle MI, which is based on centroidal distance. The initial stage encompasses the simulation of DEM mixing within a virtual environment to produce the test image stack. A novel MI model is formulated to address several problems of the previous linear model. Subsequently, a series of vision-processing algorithms are designed to measure MI by identifying the region of the particles of interest in the image. The centroid and standard deviation information is derived from the pixel distribution of the chosen particles and is refined using the moving average filter. Ultimately, the test results are showcased to validate the efficiency of the suggested algorithm.

### 2.1. Preliminary Discrete Element Simulation

DEM is the most widely used tool for the analysis of particle processes, providing substantial benefits in the exploration of granular flow dynamics [7,8,9,10,11]. DEM enables the straightforward creation and manipulation of particles, enabling the enforcement of specific operating conditions. Notably, it allows for the consideration of diverse contact models, which are vital for the precise description of particle interactions. With DEM, it is feasible to collect time series data on the kinematic and kinetic variables of individual particles. These comprehensive data can be analyzed to determine various process parameters, such as MI. Therefore, DEM simulations provide an essential understanding of the configuration and preparation of the experimental equipment prior to its actual assembly and operation. This predictive ability helps optimize the process setup, thus minimizing the time and cost associated with experimental trials. Moreover, the post-processing features of DEM simulations facilitate the production of video data of particle-mixing processes, which are indispensable for the development and validation of image-based MI calculation algorithms. The capacity to specify the colors of particles and viewing perspectives in the course of creating simulation visuals assists in generating video data, which are ideally tailored for ensuing image-processing activities. In this study, DEM simulations were conducted to leverage these benefits, providing a foundation for the real-time monitoring and control of particle-mixing processes. The simulations not only fine-tuned the experimental design but also confirmed the image-based analysis methods developed in this study.

In this study, the commercial Altair^®^ EDEM™ version 2022.2 software package was used for DEM simulations of particle mixing. The mixer used in DEM simulations is a cylindrical vessel with a diameter of 300 mm and a height of 100 mm, structured as a rotary drum. To facilitate the capture of images from external cameras, the mixing vessel was constructed of transparent acrylic. The particles involved in the mixing simulation consist of 20 mm-diameter red particles and 10 mm-diameter blue particles, both made of polyethylene. These sizes were intentionally varied to induce segregation during the mixing process [23], which improved the study of particle dynamics under differential motion conditions. The properties of the materials used in the DEM analysis are presented in Table 1.

Both particle-to-particle and particle-to-vessel contact models used the identical Hertz–Mindlin model, which is a widely used contact model in DEM for non-cohesive particulate materials [24]. Its realistic representation of particle interactions, versatility [25], compatibility with experimental data [26], fundamental role in effective medium theories [27], and improved precision and stability [28] are primary factors for its extensive application in DEM simulations. In addition to the contact force, the model also takes into account sliding and rolling friction. Cohesion and adhesion are not included in this study, as the moisture content was negligible compared to the inertial forces of the particles. The Hertz–Mindlin contact model characterizes the viscoelastic response during particle collisions. Interactions involving elastic contact are grounded in Hertzian elastic contact theory. The damping coefficient, which is nonlinear and represents the viscous term, is defined independently of the velocity of the particles and is characterized by the restitution coefficient. Given the extensive resources available on the Hertz–Mindlin contact model [5,8,20,29,30], this paper does not explore its theoretical foundation. The parameters used in the contact model are shown in Table 2. The coefficient of restitution for polyethylene and acrylic was determined through rebound experiments involving free-fall tests. Due to the experimental difficulties in directly measuring the tangential and rolling friction coefficients, the calibration approach suggested in [31] was utilized. This approach calibrates these coefficients using the observed slope of the particle bed in drum-mixing experiments. The contact model did not include cohesion or adhesion models, as there was no evidence of particle agglomeration in the particle-mixing experiments.

**Figure 1:**
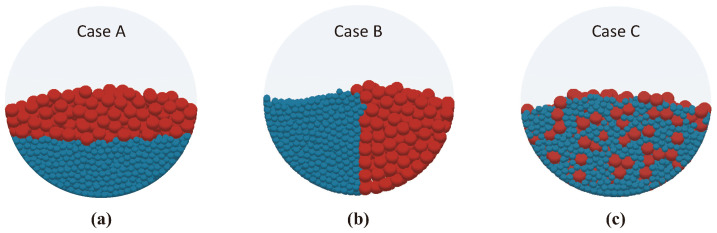
Three cases of initial arrangement of 250 large red particles and 2000 small blue particles: (**a**) Case A for horizontal division, (**b**) Case B for vertical division, and (**c**) Case C for arbitrary dispersion.

The impact of initial particle distribution on the mixing and segregation phenomena was investigated by setting three different initial configurations. These configurations were designated as Case A, Case B, and Case C. In Case A, the small and large particles were separated vertically, while in Case B, they were separated horizontally. In Case C, the particles were mixed randomly. The initial configurations were observed from the front view of the cylindrical vessel as illustrated in Figure 1. For each of the three cases, 250 large red particles and 2000 small blue particles were generated.

Table 3 presents the parameters used in the DEM simulations. The mixer was configured to rotate at a speed of five revolutions per minute (rpm). This rotational speed corresponds to a Froude number of 0.014 and, with the particles filled to 50% (filling degree of 0.5), induces a rolling bed state within the drum. This state is classified as a mixing mode in [31,32], where similar conditions have been shown to facilitate effective particle mixing. The size of the computation domain was modified to encompass both the vessel and the particles. The search algorithm for neighboring contact detection required the computation domain to be discretized into cubic background cells. The cell size was determined to be 25 mm, which is 2.5 times the maximum diameter of a particle, to optimize the computational efficiency [33]. The explicit Euler method was used for numerical integration, carried out in 3,375,000 time steps, with a total simulation time of 60 s. The images, processed and stored from the DEM software, were generated to aid in the development and evaluation of the vision-based MI calculation algorithm.

**Figure 2:**
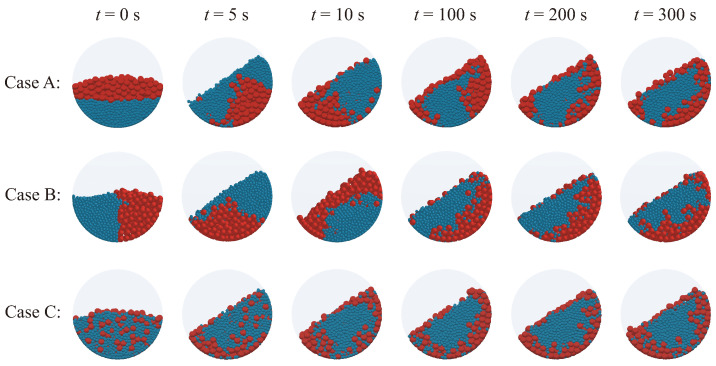
DEM simulation results for Case A, Case B, and Case C at 0 s, 5 s, 10 s, 100 s, 200 s, and 300 s.

Figure 2 shows the post-processed graphics of the DEM simulation results for the three initial particle conditions shown in Figure 1. A frontal viewpoint of the circular face of the cylindrical vessel was selected for observation. To observe the early-stage transient response, three images were extracted: the initial-time image and images at 5 and 10 s. In addition, to examine the response after a sufficient process time, three more images were chosen: images at 100, 200, and 300 s. According to published studies [23], smaller particles tend to migrate toward the center, while larger particles move away due to differences in size, resulting in radial segregation of particles. During the drum-mixing operation, radial segregation occurs as particles of varying sizes or densities are sorted into separate radial layers within the drum. This segregation is due to differences in the movement and settling characteristics of the particles as the drum rotates [34]. Remarkably, even in Case C, which begins in a thoroughly mixed state, radial segregation eventually manifests, similarly to the other scenarios, as the mixer operation continues.

It is important to note that DEM exhibits certain constraints in particle-mixing simulations. DEM simulations are computationally demanding, especially when dealing with systems that involve a large number of particles. The computational cost increases with the number of particles and the complexity of their interactions [35]. This makes the expansion of DEM simulations to industrial-scale mixing processes difficult, as they demand substantial computational resources, thus restricting their feasibility in large-scale industrial applications [36]. Furthermore, the precision of DEM simulations is significantly dependent on the appropriate choice and calibration of the contact models and parameters. Inaccuracies in these models can lead to erroneous simulation results [37].

### 2.2. Mixing Index Formulation

Various mixing index models have been proposed to quantify the state of the particle mixture [8,14]. In this particular study, a centroid-based MI is used instead of using the widely used GLCM method or the Lacey index. This MI model offers a significant advantage over the other methods in terms of computation time complexity. While the GLCM method and the Lacey index require pixel randomization, $O(N^{2})$ computation time complexity to calculate contacts with adjacent particles in an *N*-particle system, or partitioning into subdomains, the centroid-based MI approach offers a reduced time complexity of O(N). The centroid-based MI measures the degree of mixing by calculating the difference between the centroids of distinct particle groups. The principle behind this MI is that the centroidal distance between the particle groups decreases as the mixing process progresses. In vision-based particle differentiation by color, the computation relies on the centroid of pixel distribution, rather than a mass center, because the density of the particles is not directly measurable via computer vision. This method of calculating MI is commonly known as the average height method. It involves measuring the average height of particles in a vertically fixed conic mixer, such as a spouted bed, in a one-dimensional sense [8]. When expanded to three dimensions, the generalized mean mixing index (GMMI) is used to denote a centroid-based mixing index [9]. Specifically, this paper improved the previous linear MI used to quantify the mixing of binary solid particles [20,21] and fluid particles [22], which can be written as:

(1) $$MI_{\mathrm{linear}}=1-\frac{d}{d_{0}}$$

where *d* represents the centroidal distance between two groups of particles and $d_{0}$ denotes its initial value. This linear model operates on the principle that MI is zero at the start of the mixing process and increases towards unity as the mixing progresses from an inhomogeneous state to a completely homogeneous state. The linear relationship between the MI and the centroidal distance is advantageous because of its simplicity and computational efficiency. However, during the initial stages of mixing, the centroidal distance changes significantly, causing the MI to increase very sensitively, whereas in well-mixed states, the rate of change slows down as the centroidal distance converges to zero, making the MI less sensitive. This linear model maintains constant sensitivity, thus failing to accurately reflect such an inconstant sensitivity. Furthermore, during the mixing process, the centroidal distance could increase beyond the initial state, resulting in a negative MI. In addition, the MI may oscillate significantly with each rotation cycle in the case of rotary mixers. The most significant drawback of this MI model arises in cylindrical vessels when radial particle segregation occurs. It is important to note that although the particles may be radially separated, the centroids of the central and peripheral particles may not differ significantly. This might result in an MI value approaching one, which could be misleading, as it implies thorough mixing of the particles, whereas substantial radial segregation actually exists.

This research introduces a novel MI model that inherits the computational efficiency of linear MI in the form of Equation (1) while addressing its previously mentioned shortcomings through the development of a nonlinear MI model. Firstly, to address negative MI and sensitivity, a sigmoid function is introduced, which is defined as:

(2) $$y=\frac{1}{1+e^{-x}}$$

The sigmoid function is characterized by a smooth, symmetric curve centered at the point (0.5, 0.5), with its values confined within the range 0 to 1. As the functional value approaches the lower or upper bound, its sensitivity decreases compared to the symmetry point. Specifically, the values of the sigmoid function are approximately 0.0067 at x=−5 and 0.9933 at x=5, indicating sufficient saturation around x=±5.

The following MI formula modifies the sigmoid function, as presented in Equation (2), to meet the particle MI requirements. This modification includes sequential transformations of the original sigmoid curve, involving compression by a factor of 10 along the *x*-axis, a shift of 0.5 units in the positive *x*-direction, and a reflection about the horizontal line at y=0.5:

(3) $$MI_{\mathrm{sigmoid}}=1-\frac{1}{1+e^{-10\left(\frac{d}{d_{c}}-\frac{1}{2}\right)}}$$

where $d_{c}$ denotes the characteristic distance, defined as the centroidal distance at which particles of two different types occupy half of the cylindrical vessel each, arranged in a semicircular pattern. Hence, the normalized centroidal distance $\frac{d}{d_{c}}$ as applied in Equation (3) depends on the radius of the vessel instead of the initial conditions of particle filling, unlike $\frac{d}{d_{0}}$ in Equation (1).

**Figure 3:**
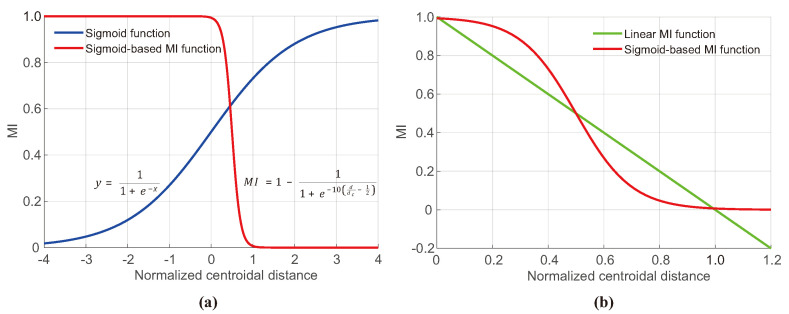
Graphs showing (**a**) the sigmoid function and its adapted form for MI estimation, and (**b**) comparisons between linear and sigmoid-based nonlinear MI functions.

As illustrated in Figure 3a, the plot of Equation (3) shows that this modified MI is unity when *d* approximately equals 0, and as *d* nears $d_{c}$, MI approaches 0. Furthermore, as shown in Figure 3b, the sigmoid-based MI remains non-negative even when the normalized centroidal distance exceeds 1, also featuring a decreased sensitivity as *d* approaches 0, unlike the linear MI expressed as Equation (1).

To address the issue of incorrect high MI predictions in cases of radial segregation, Equation (3) is further modified by multiplying it with a standard deviation weighting factor as below:

(4) $$MI=\left[1-\frac{1}{1+e^{-10\left(\frac{d}{d_{c}}-\frac{1}{2}\right)}}\right]\left[1-\frac{1}{1+e^{-10\left(\frac{\Delta \sigma }{\sigma_{c}}-\frac{1}{2}\right)}}\right]\;$$

where Δσ denotes the difference in standard deviation of the two particle groups, and $\sigma_{c}$ represents its characteristic value depending on the vessel geometry like $d_{c}$. In this study, $\sigma_{c}$ is set to $\frac{d_{c}}{2}$ for a cylindrical mixing vessel. The weighting factor for the standard deviation as expressed in the second set of square brackets is defined identically to the $MI_{\mathrm{sigmoid}}$ in Equation (3) while replacing the normalized centroidal distance $\frac{d}{d_{c}}$ with the normalized standard deviation difference $\frac{\Delta \sigma }{\sigma_{c}}$. At Δσ=0, the weighting factor is approximately 1, decreasing to 0 as Δσ approaches $\sigma_{c}$. Therefore, when the difference in standard deviation between the two groups of particles is small, the MI value from Equation (3) experiences minor adjustments. However, with increasing standard deviation difference, there is a significant weighting effect to decrease MI. This adjustment of MI through the weighting factor prevents the overestimation of MI during radial segregation in a cylindrical container.

Furthermore, a moving average filter is applied to reduce the occurrence of MI data fluctuations observed in rotary mixers [38]. This filter operates by calculating the mean values from a specified size of the moving data buffer, thus efficiently stabilizing variations in centroid-driven MI measurements.

### 2.3. Image-Processing Algorithm for Mixing State Monitoring

**Figure 4:**
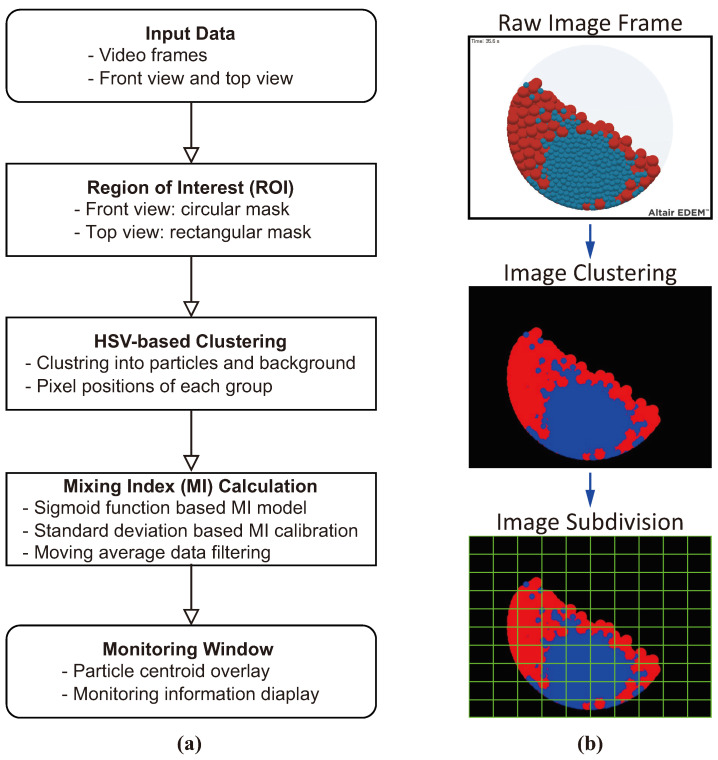
Algorithm for MI computation from vision data: (**a**) flowchart of image processing and (**b**) processed image samples with an optional stage for subdomain division.

A flow chart of the computer vision algorithm used to calculate the MI from video frames that monitor particles in a mixing vessel is presented in Figure 4a. Initially, the background is removed from the image, and the region of interest (ROI), including only the mixer geometry, is extracted. Circular and rectangular masks are used to specify the ROI for the front view and top view, respectively, of the cylindrical vessel. The particles are clustered by extracting colors using hue saturation value (HSV) filtering. This is illustrated in the second stage in Figure 4b. Pixels not designated as particles are considered the background and filled with single hues of color, such as black or white. If subdomain partitioning, as in the Lacey index method, is required, the final step in Figure 4b should be additionally performed. It is worth noting that this study computes MI using a non-sampling method that does not require subdomain partitioning. The pixel location information of the color clusters extracted through HSV filtering is used to calculate the centroid and standard deviation of each particle cluster. The MI for each frame can be quantified using Equation (4). A moving average filter is utilized to generate a trend line over time from the highly fluctuating MI data. For this study, the data buffer size for filtering was set to 150.

**Figure 5:**
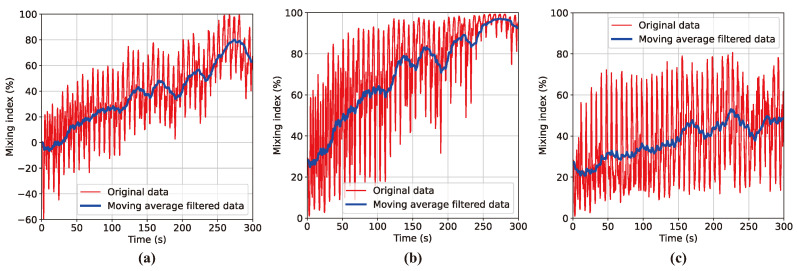
MI–time curve obtained from DEM simulation results for Case A with three different MI models: (**a**) linear MI model, (**b**) sigmoid-based MI model, and (**c**) sigmoid-based MI model incorporating standard deviation correlation factor.

The MI–time curves shown in Figure 5 were obtained by applying the vision process illustrated in Figure 4 to the post-processed image stack from the results of the DEM mixing simulation for the initial particle distribution of Case A. This figure demonstrates the advantages of the newly proposed MI model by comparing the MI plots obtained using the traditional linear MI model represented by Equation (1), the nonlinear MI model using a transformed sigmoid function defined by Equation (3), and the MI based on the sigmoid function calibrated with a standard deviation weighting factor expressed by Equation (4). Figure 5a shows the curve obtained by the linear MI model that adopts the initial centroidal distance $d_{0}$ as the normalization factor. However, this model encounters the problem of negative MI values, which was previously highlighted. This negative MI arises when the vessel rotation causes particle dispersion shortly after the mixing process begins. This dispersion leads to *d* exceeding $d_{0}$, resulting in a negative MI. As the mixer operation progresses, radial segregation becomes increasingly evident, yet the MI value escalates excessively to approximately 80%, failing to provide an accurate representation of the mixing state. Introducing the modified sigmoid function resolves the issue of negative MI values, but MI still rises to 90%, inaccurately quantifying radial segregation. Figure 5c shows that the incorporation of a standard deviation weighting factor into the MI model can reflect radial segregation, the final MI being saturated around 50%. The three graphs in Figure 5 exhibit significant fluctuations in the MI calculation results. However, data filtering helps to identify the trend line more clearly, even in the midst of these fluctuating data.

**Figure 6:**
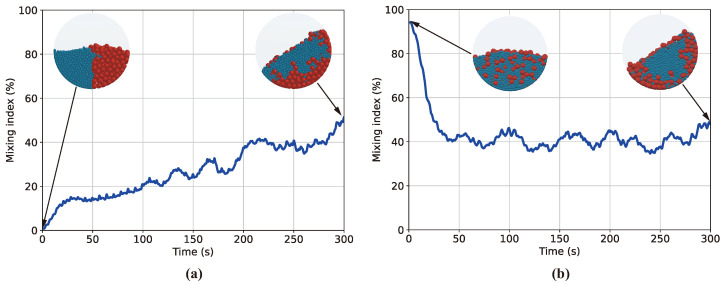
Curves of MI over time derived from DEM simulation results using computer vision processes for (**a**) Case B and (**b**) Case C, with included images showing particle distribution at initial and final states.

Figure 6 shows the MI–time curves obtained from the DEM simulation results for Case B and Case C. Figure 6a illustrates that the MI–time curve starts at nearly 0 due to the highly separated initial particle configuration and eventually saturates in the mid-40% range due to persistent radial particle segregation. Figure 6b further supports the versatility and precision of the image-processing algorithm and the MI model proposed in this study. Not only does it quantify radial segregation but it also demonstrates high precision in quantifying homogeneous mixing, as the MI exhibits values above 90% for a highly mixed initial condition. In Figure 6b, the MI curve quantitatively infers that segregation occurs due to particle size differences, even if the mixer operation starts in a randomly distributed state.

## 3. Experimental Result and Discussion

### 3.1. Real-Time Data Acquisition

**Figure 7:**
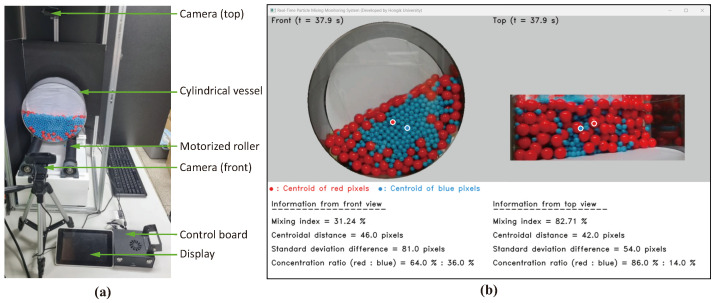
Experimental setup: (**a**) apparatus and (**b**) real-time MI monitoring display.

Figure 7 shows the experimental setup and monitoring screen of an experimental apparatus. The system uses the vision-processing algorithm described in Section 2.2 and the MI model explained in Section 2.3 to monitor the real-time mixing of the particles. The mixing drum is made of acrylic and measures 300 mm in diameter and 100 mm in height. The desktop ball mill (model LM-BD4530 produced by LKLABKOREA Inc., based in Namyangju-si, Republic of Korea) rotates the drum via a motorized roller, with an adjustable rotation speed of up to 350 rpm. Two webcams (model C920e manufactured by Logitech International S.A., headquartered in Lausanne, Switzerland) are installed to monitor the mixing state from both front and top views. These cameras can record video at 30 fps with a high-definition resolution of 1920 × 1080 pixels. The control board uses the Raspberry Pi 5 model, which is manufactured by the Raspberry Foundation in Cambridge, UK. It is an SBC that runs on the Linux operating system and is equipped with a quad-core ARM Cortex-A76 CPU and 8 GB of memory. A 7-inch light-emitting diode (LED) display was installed to allow the real-time monitoring of the vision-processing results and process variables such as MI. The user interface to monitor the mixing state of the particles is shown in Figure 7b. The monitoring window on the upper part displays images with extracted ROIs and overlay markers indicating the centroidal positions of each particle group distinguished by color. In the lower part of the window, key process variables such as MI, centroidal distance, standard deviation difference, and particle concentration ratio are updated in real time. The implementation of vision processing and the monitoring interface employs in-house Python code. Furthermore, data from the process are recorded in real time into a file in comma-separated values (CSV) format, which is utilized to produce MI curves and corresponding result graphs. It is important to recognize that this technique cannot be used with opaque vessels because it depends on RGB camera footage and computer vision methods to determine the degree of particle mixing in real time.

**Figure 8:**
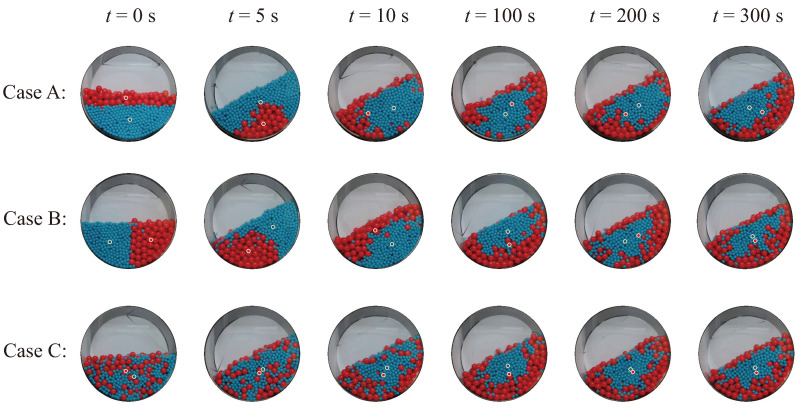
Experimental results for Case A, Case B, and Case C at 0 s, 5 s, 10 s, 100 s, 200 s, and 300 s.

Figure 8 shows the front view images obtained from the process-monitoring window. These images were captured during real-time process-monitoring experiments that featured the three initial particle conditions illustrated in Figure 1. Circular marks overlaid on the vessel image indicate the current positions of the centroids for each particle group. The images were extracted at the same time points as those shown in Figure 2, which shows the results of the EDEM simulations. As depicted in Figure 8, radial segregation phenomena due to particle size differences were commonly observed in three experimental cases, consistent with the predictions from the simulation results.

### 3.2. Data Analysis and Discussion

**Figure 9:**
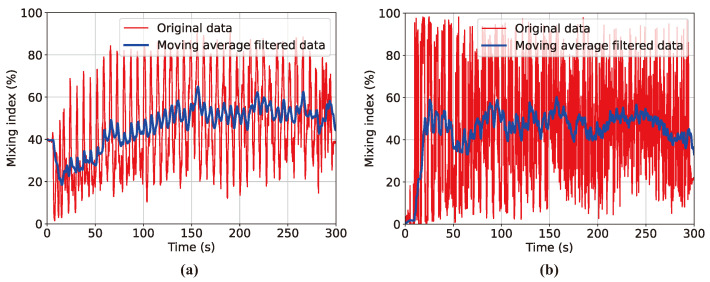
MI curves obtained from real-time monitoring system through image processing and moving average data filtering of video frames for (**a**) front view and (**b**) top view for Case A.

Figure 9 displays the MI curves resulting from the image processing of a 300 s mixing experiment in the initial particle configuration of Case A. The experiment was carried out using the real-time particle-mixing monitoring system shown in Figure 7. The MI curve, calculated from the front view, shows data fluctuations similar to those observed in the Figure 5c simulation results. The MI curve exhibits more frequent and larger amplitude fluctuations when viewed from above compared to the front view. This is due to the radial segregation effect, in which the concentration ratios of particles on the drum’s top surface change significantly over time as a result of its rotation. For example, Figure 7b shows a composition ratio of 86% red particles to 14% blue particles, which is markedly different from the 64% to 36% ratio observed from the front view. At times, the top surface is entirely dominated by one color, resulting in considerable variations in the initial MI data.

**Figure 10:**
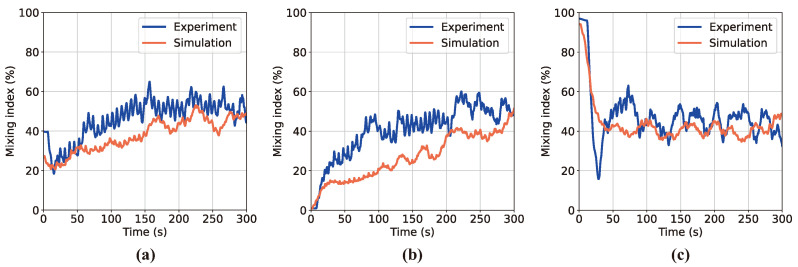
Comparison of MI curves from real-time mixing experiments and DEM simulations across three scenarios: (**a**) Case A, (**b**) Case B, (**c**) Case C.

Figure 10 presents a comparison of the MI graphs derived from image processing of the experimental and DEM simulation results for the three initial conditions depicted in Figure 1. During the analysis of experimental data, it should be emphasized that common issues such as perspective effects, lighting glare, and reflections from particles on the vessel walls may introduce computational inaccuracies in the clustering phase of particle groups. In contrast, simulations offer a more direct approach to adjusting and controlling image colors and compositions, simplifying the color-based clustering process with fewer computational errors. The analysis of moving average lines from both the experiment and simulation reveals slight variations in the agitation levels. However, the consistent trends observed confirm the reliability of the real-time particle-mixing monitoring system developed in this study.

**Figure 11:**
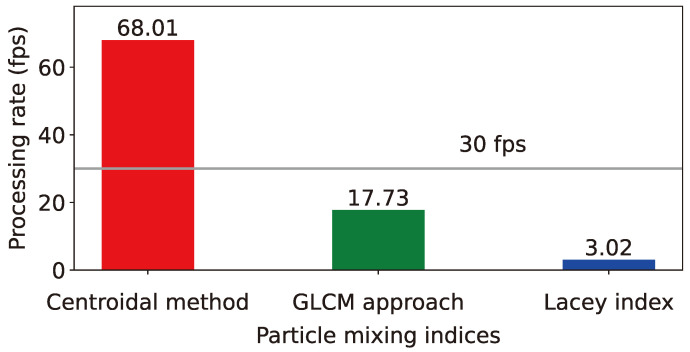
Comparison of image-processing rates in frame per second using three different techniques for calculating particle-mixing index, namely, the centroid-based method, GLCM approach, and Lacey index.

Figure 11 displays a bar graph that contrasts the image-processing speeds of three representative particle MI calculation techniques: the centroid-based method, the GLCM approach, and the Lacey method, performed on the affordable and simple-to-install Raspberry Pi 5 instead of expensive high-performance workstations. The results reveal that the centroid-based method, introduced in this research, attains an image-processing speed of 68.01 fps for images of 640 × 480 pixel resolution, markedly surpassing the 17.73 fps achieved by the GLCM approach and the 3.02 fps achieved by the Lacey method, underscoring its enhanced computational performance. Considering that standard monitoring cameras generally record at 30 fps, the centroid-based method alone meets the requirements for real-time image processing to compute process parameters such as MI. Due to the substantial computational demands of traditional MI techniques, previous studies on determining mixing index from particle mixture images have mainly focused on analyzing pre-recorded videos [13,16,17,18] rather than real-time monitoring. The significance of this study lies in developing an improved MI model that can reflect particle segregation while being computationally light, using cost-effective SBCs and webcams, and implementing a practical monitoring system prototype capable of real-time particle process monitoring.

## 4. Conclusions

This study successfully developed and validated a real-time vision system capable of monitoring the mixing process of polymeric particles in a rotary drum mixer using an improved centroid-based model to calculate the mixing index. The innovative approach significantly reduced the computational load compared to traditional methods, such as Lacey index-based or GLCM methods, thereby enabling real-time processing at image-processing rates suitable for practical industrial applications. Cross-validation of the system using simulations from the discrete element method affirmed the accuracy and dependability of the proposed vision-based mixing monitoring system. The capability of the system to calculate the mixing index efficiently under real operational conditions demonstrates its potential to enhance process control and quality assurance in the manufacturing of polymer products. Future research will focus on further optimizing computational algorithms to handle higher complexities in mixing scenarios and exploring the scalability of technology for larger industrial applications. In addition, integrating this technology with IoT devices for improved data collection and analysis could pave the way for smarter manufacturing environments. This research not only provides a robust tool for enhancing the efficiency of polymer mixing processes but also contributes to the broader field of materials engineering by introducing a novel, real-time analytical method that can be adapted to various industrial processes.

## Figures and Tables

**Table 1:** Properties of polymer balls and cylindrical vessel.

Property	Ball (Polyethelene)	Vessel (Acrylic)
Mass density ($\mathrm{kg}/m^{3}$)	931	1180
Modulus of elasticity (GPa)	0.76	1.70
Poisson’s ratio	0.25	0.37

**Table 2:** Parameters for Hertz–Mindlin contact model.

Parameter	Ball–Ball	Ball–Vessel
Coefficient of restitution	0.5	0.5
Coefficient of sliding friction	0.25	0.25
Coefficient of rolling friction	0.1	0.1

**Table 3:** DEM simulation parameters.

Parameter	Value
Drum rotation speed (rpm)	5.0
Computational time step (µs)	17.78
Simulation time (s)	60
Number of iterations	3,375,000
Computation domain size (${\mathrm{mm}}^{3}$)	306×306×202
Unit length of background cells (mm)	25.0
Number of cubic cells	5625

## Data Availability

Data are contained within the article.

## Nomenclature

*d*	centroidal distance between two particle groups, pixels
$d_{0}$	initial centroidal distance, pixels
$d_{c}$	characteristic centroidal distance, pixels
$MI_{\mathrm{linear}}$	linear mixing index
$MI_{\mathrm{sigmoid}}$	sigmoid-based MI function
*N*	number of total particles
*O*	Big O notation
Creek symbols	
Δσ	difference in standard deviation of two particle groups, pixels
$\sigma_{c}$	characteristic standard deviation, pixels
Abbreviations	
CSV	comma-separated values
DEM	discrete element method
FDM	fused deposition manufacturing
fps	frames per second
GLCM	gray-level co-occurrence matrix
GMMI	generalized mean mixing index
HSV	hue saturation value
LED	light-emitting diode
MI	mixing index
RGB	red-green-blue
ROI	region of interest
SBC	single-board computer
SI	segregation index
